# Effects of a luteinizing hormone-releasing hormone agonist on cognitive, sexual, and hormonal functions in patients with prostate cancer: relationship with testicular and adrenal androgen levels

**DOI:** 10.1186/s12610-015-0019-y

**Published:** 2015-04-06

**Authors:** Kohei Okamoto, Yositaka Sekine, Masashi Nomura, Hidekazu Koike, Hiroshi Matsui, Yasuhiro Shibata, Kazuto Ito, Kazuhiro Suzuki

**Affiliations:** Department of Urology, Gunma University Graduate School of Medicine, 3-39-22 Showa-machi, Maebashi, 371-8511 Gunma Japan

**Keywords:** Prostate cancer, LH-RH agonist, Sexual and hormone functions, Cognitive function, Androgens

## Abstract

**Purpose:**

To assess the cognitive and sexual/hormonal functioning of prostate cancer patients treated with a luteinizing hormone-releasing hormone (LH-RH) agonist, and the relationships thereof with adrenal and residual testicular androgen levels.

**Materials and methods:**

Previously, we reported the effect of a luteinizing hormone-releasing hormone (LH-RH) agonist on testicular and adrenal androgen production in patients with prostate cancer. A 6-month treatment with an LH-RH agonist significantly reduced testicular androgens by 90–95% and adrenal androgens by 26–40%. This study evaluated the changes in cognitive and sexual/hormonal functions in the same cohort using the Mini-Mental State Evaluation (MMSE) and Expanded Prostate Cancer Index Composite (EPIC) questionnaire, respectively. In addition, the associations of each function with the serum testosterone (T), dihydrotestosterone (DHT), estradiol (E2), dehydroepiandrosterone-sulfate (DHEA-S), dehydroepiandrosterone (DHEA), androstenedione (A-dione), and cortisol levels were studied.

**Results:**

Cognitive functions did not change significantly during the treatment. Sexual functions were relatively low before treatment and worsened significantly after 6 and 12 months of treatment. Interestingly, sexual bothers were improved with the treatment. The treatment significantly worsened hormonal functions and bothers. Regarding specific items in the hormonal domains, hot flashes and body weight changes were the main effects of worsened hormonal function. Low levels of T and E2 and high levels of A-dione were associated with low MMSE scores at 6 months. Regarding sexual and hormonal functions, A-dione, E2, T, cortisol, and DHEA-S were associated with poorer functioning and bother. Especially, low T levels and high E2 levels were the most significant factors associated with worse sexual and hormonal bothers.

**Conclusion:**

The LH-RH agonist monotherapy worsened sexual and hormonal functions and hormonal bothers, but not sexual bothers or cognitive functions. The changes in these functions were related to the testicular and adrenal androgens levels.

## Introduction

Prostate cancer is an androgen-dependent cancer. Long-term androgen-deprivation therapy (ADT) is the standard therapy for metastatic prostate cancer and is administered in localized or locally progressive disease concomitant with radiation therapy for the short or long term [1]. ADT has several adverse effects [2,3]. ADT with luteinizing hormone-releasing hormone (LH-RH) analogues is used frequently for medical castration. Previously, we reported the effect of an LH-RH agonist on reducing serum adrenal androgen levels 6 and 12 months after initiating treatment [4]. We also identified immunoreactive LH receptors in the reticular layer of the adrenal glands and speculated that the LH-RH agonist therapy reduced adrenal androgen synthesis via reduced LH levels [4].

This study assessed the adverse effects of ADT in the same cohort, focusing on cognitive and sexual/hormone functions. We evaluated the changes in the health-related quality of life (QOL) and cognitive functions using the Expanded Prostate Cancer Index Composite (EPIC) questionnaire [5,6] and Mini-Mental State Evaluation (MMSE) [7]. In addition, the changes in the QOL and cognitive function parameters were compared with serum levels of testicular and adrenal androgens.

## Materials and methods

### Patients

Previously, we evaluated the serum testicular and adrenal androgen levels in 47 patients with prostate cancer treated via 6-month neoadjuvant ADT with radiation therapy, followed by adjuvant ADT (this treatment strategy was reported previously by our study group [8]). ADT was given as monotherapy; we prescribed the LH-RH agonist leuprolide. Forty-five of the forty-seven patients had full QOL and cognitive function test data and were included in this study. Table 1 shows the clinical characteristics of the patients analyzed. Their ages ranged from 61 to 75 years, with a mean age of 67.5 years. The mean pretreatment prostate-specific antigen (PSA) level was 13.0 ng/mL. Using the D’Amico risk stratification, 16 patients (35.6%) were categorized in the intermediate-risk group and 29 patients (64.4%) in the high-risk group. This study was approved by the Ethics Committee of the Gunma University Faculty of Medicine, and written consent was obtained from the enrolled patients.

**Table 1:** **Clinical characteristics of the enrolled patients**

n	45	
Age (±SD)	67.5 ± 3.5	
PSA (±SD)	13 ± 10.7	
G.S.	6	1
	7	28
	8	9
	9	7
T	T1c	12
	T2a	1
	T2b	7
	T2c	7
	T3a	17
	T3b	1
Risk group	Intermediate	16
	High	29

PSA: prostate specific antigen.

G.S. Gleason score.

T:T category of TNM Classification of Malignant Tumours 7th Edition.

Risk group: D’Amico risk stratification.

### QOL questionnaires and cognitive function test

The QOL was assessed using the EPIC questionnaire [5] before and 6 and 12 months after initiating the LH-RH agonist treatment. In this study, the sexual and hormonal domains were analyzed. For the sexual domain, the mean summary scores and subscale scores; *i.e*., sexual function and bother, were assessed. For the hormonal domain, the score changes for all items in the hormonal domain were studied, as well as the mean summary and subscale scores. Statistical significance was determined using the χ^2^ test. Cognitive function was assessed using Mini-Metal State Examination [7] and the same time points as the QOL assessment. The examination was administered by one medical technologist (R.S.).

### Blood samples and measuring hormone levels

As described elsewhere [4], serum testosterone (T), dihydrotestosterone (DHT), estradiol (E2), dehydroepiandrosterone (DHEA), and androstenedione (A-dione) were measured using liquid chromatography-mass spectrometry (LC-MS). DHEA-sulfate (DHEA-S) was measured using a chemiluminescence enzyme immunity assay. In this study, we also measured the serum cortisol levels using LC-MS. Blood samples were taken before and 6 and 12 months after initiating the LH-RH treatment. The changes in hormone levels at each evaluation point are shown in Table 2. As mentioned earlier, the serum levels of T, DHT, E2, DHEA-S, DHEA, and A-dione decreased significantly during the treatment, while the cortisol levels did not differ significantly.

**Table 2:** **Changes in the serum hormone levels in prostate cancer patients treated with a luteinizing hormone-releasing hormone agonist**

		**PRE**	**6MO**	**12MO**	**Statistics**
T	Mean (ng/dL)	378.8 ± 145.1	9.6 ± 5.0	7.7 ± 4.6	P < 0.01 PRE vs, 12MO
	Percentile change		-97.5%	-98.0%	
DHT	Mean (pg/mL)	420.2 ± 200.2	21.6 ± 12.2	20.8 ± 11.2	P < 0.01 PRE vs 6MO, 12MO
	Percentile change		-94.9%	-95.0%	
E2	Mean (pg/mL)	16.2 ± 5.2	1.3 ± 1.0	1.4 ± 1.0	p < 0.01 PRE vs 6MO, 12MO
	Percentile change		-92.2%	-91.3%	
DHEA-S	Mean (*μ*g/dL)	144.9 ± 66.6	107.2 ± 48.6	103.5 ± 54.3	P < 0.01 PRE vs 6MO, 12MO
	Percentile change		-26.1%	-28.6%	
DHEA	Mean (ng/mL)	1.85 ± 0.91	1.38 ± 0.67	1.29 ± 0.65	P < 0.01 PRE vs 6MO, 12MO
	Percentile change		-25.1%	-30.0%	
A-dione	Mean (pg/mL)	384.0 ± 133.5	231.7 ± 109.4	227.8 ± 119.7	p < 0.01 PRE vs 6MO, 12MO
	Percentile change		-39.7%	-40.7%	
cortisol	Mean (ng/mL)	89.9 ± 26.7	94.9 ± 27.4	81.4 ± 23.4	Not significant
	Percentile change		5.4%	-10.4%	

PRE: pretreatment 6MO: 6 months after initiation of LH-RH agonist treatment.

12MO: 12 months after initiation of LH-RH agonist treatment.

T: testosterone, DHT: dihydrotestosterone, E2; estradiol, DHEA-S: dehydroepiandrosterone-sulfate.

DHEA: dehydroepiandrosterone.

Percentile change: changes in comparison with pretreatment levels.

The percentile change is in comparison with the pretreatment levels. Values are expressed as means ± SD (standard deviation).

### Evaluation of sexual, hormonal, and cognitive functions according to age-adjusted hormone levels

To evaluate the association between the hormonal environments and QOL and MMSE scores at each observation point, a multivariate analysis was performed. As dependent variables, the MMSE scores, and sexual and hormonal subscale scores were adopted (0 ≥ mean, 1 < mean). Differential values were the hormone levels and age at each observation point. Using stepwise multiple regression analysis, significant predictive factors were estimated. The statistical analysis was performed using SPSS ver. 19.0 (SPSS, Chicago, IL, USA).

## Results

**Figure 1:**
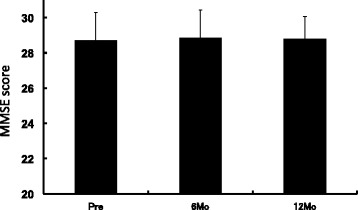
**Changes in MMSE scores.** Values are expressed as means ± SD. PRE: pretreatment, 6Mo: 6 months after initiation of treatment, 12Mo: 12 months after initiation of treatment.

The changes in the MMSE scores are shown in Figure 1. The mean pretreatment score was 28.7, and no significant difference was observed after 6 or 12 months of treatment. Stratification of the patients based on pretreatment scores; *i.e*., ≥ mean or < mean, showed no significant difference in the mean scores during treatment in either the high or low baseline score groups (data not shown).

**Figure 2:**
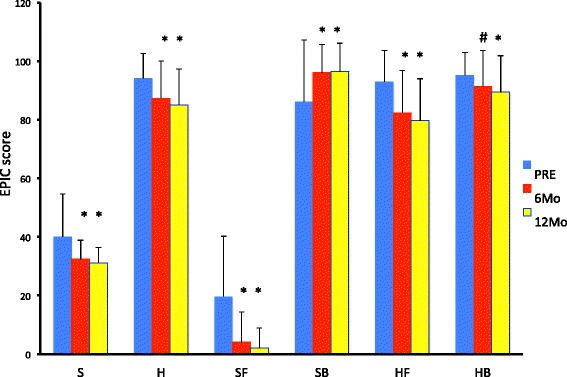
**Mean sexual and hormone domain scores including summary score and subscale scores.** Values are expressed as means ± SD, and pretreatment values are compared with those at each post-treatment sampling point. A difference was considered to be significant if the *p*-value was less than 0.05 (# *p* < 0.05, * *p* < 0.01). EPIC: Expanded Prostate Cancer Index Composite. PRE: pretreatment, 6Mo: 6 months after initiation of treatment, 12Mo: 12 months after initiation of treatment **Summary score**: S; sexual summary score including sexual function and sexual bother scores. H; hormone summary score including hormone function and hormone bother scores. **Subscale scores**: SF; sexual function, SB; sexual bother, HF; hormonal function, HB; hormone bother.

Figure 2 shows the changes in the sexual and hormonal scores. The summary scores of both the sexual and hormonal domains worsened significantly during treatment. Regarding the subscale scores, the sexual function scores worsened significantly, whereas the sexual bother scores improved significantly. By contrast, the scores for both hormonal function and bother worsened significantly.

Tables 3 and 4 summarize the frequency of each item for hormonal function and bother, respectively. The functional and bother changes; *i.e*., hot flashes, were worsened significantly during treatment. Significant body weight gains were observed.

**Table 3:** **Distribution of EPIC hormone function domain items before and after 6 and 12 months of androgen-deprivation therapy**

	**More than once a day**	**About once a day**	**More than once a week**	**About once a week**	**Rarely or never**
Hot flashes*					
Pre	4.4%	4.4%	0.0%	6.7%	84.4%
6Mo	33.3%	13.3%	6.7%	0.0%	46.7%
12Mo	44.4%	8.9%	2.2%	4.4%	40.0%
Breast tenderness					
Pre	2.2%	0.0%	0.0%	0.0%	97.8%
6Mo	0.0%	2.2%	0.0%	0.0%	97.8%
12Mo	0.0%	0.0%	2.2%	2.2%	95.6%
Feel depressed					
Pre	0.0%	2.2%	2.2%	13.3%	82.2%
6Mo	0.0%	2.2%	4.4%	8.9%	84.4%
12Mo	0.0%	0.0%	4.4%	13.3%	82.2%
Lack of energy					
Pre	0.0%	6.7%	6.7%	22.2%	64.4%
6Mo	0.0%	4.4%	11.1%	15.6%	68.9%
12Mo	2.2%	6.7%	15.6%	15.6%	60.0%
	Gained 5 kg or more	Gained less than 5 kg	No change	Lost less than 5 kg	Lost 5 kg or more
Change in body weight*					
Pre	0.0%	2.2%	93.3%	4.4%	0.0%
6Mo	2.2%	31.1%	60.0%	6.7%	0.0%
12Mo	2.2%	35.6%	57.8%	4.4%	0.0%

*p < 0.001.

**Table 4:** **Distribution of the EPIC hormone bother domain items before and after 6 and 12 months of androgen-deprivation therapy**

	**No such symptom**	**No problem**	**Very small problem**	**Small problem**	**Moderate problem**	**Big problem**
Hot flashes*						
Pre	80.0%	6.7%	11.1%	2.2%	0.0%	0.0%
6Mo	46.7%	11.1%	20.0%	8.9%	13.3%	0.0%
12Mo	31.1%	15.6%	17.8%	20.0%	8.9%	6.7%
Breast tenderness/enlargement						
Pre	95.6%	2.2%	0.0%	0.0%	0.0%	2.2%
6Mo	93.3%	4.4%	2.2%	0.0%	0.0%	0.0%
12Mo	84.4%	8.9%	4.4%	2.2%	0.0%	0.0%
Loss of body hair						
Pre	91.1%	6.7%	0.0%	0.0%	2.2%	0.0%
6Mo	86.7%	6.7%	6.7%	0.0%	0.0%	0.0%
12Mo	66.7%	15.6%	8.9%	6.7%	2.2%	0.0%
Feeling depressed						
Pre	66.7%	8.9%	17.8%	4.4%	2.2%	0.0%
6Mo	62.2%	17.8%	8.9%	6.7%	4.4%	0.0%
12Mo	66.7%	17.8%	8.9%	6.7%	0.0%	0.0%
Lack of energy						
Pre	57.8%	11.1%	15.6%	15.6%	0.0%	0.0%
6Mo	55.6%	17.8%	13.3%	8.9%	4.4%	0.0%
12Mo	46.7%	15.6%	28.9%	4.4%	2.2%	2.2%
Change in body weight						
Pre	84.4%	11.1%	4.4%	0.0%	0.0%	0.0%
6Mo	57.8%	17.8%	15.6%	4.4%	2.2%	2.2%
12Mo	62.2%	20.0%	11.1%	4.4%	0.0%	2.2%

*p < 0.001.

Finally, we studied the associations of cognitive function, sexual, and age-adjusted hormonal functions with serum hormone levels. Low MMSE scores were associated with low E2 and cortisol and high A-dione levels at 6 months. None of the pretreatment hormone levels or decreased hormone levels were significantly related to the scores during the treatment. Regarding the sexual and hormonal function and bother scores, A-dione, E2, T, cortisol, and DHEA-S were associated with poorer functioning and bother at each observation point, as shown in Table 5. Low T levels were the factors most significantly associated with worsened sexual and hormonal bother at 6 months.

**Table 5:** **Association between hormone levels and the MMSE and EPIC scores: multivariate analysis**

MMSE					
6Mo hormone levels		B	Odds ratio	95% CI	p
	E2	-1.47	0.23	0.073-0.724	0.012
	A-dione	0.021	1.02	1.006-1.036	0.006
	cortisol	-0.059	0.94	0.898-0.989	0.016
EPIC					
Hormone levels and QOL at each observation point					
PRE					
Sexual bother	A-dione	-0.02	0.98	0.964-0.997	0.019
6Mo					
Sexual bother	E2	1.57	4.81	1.086-21.33	0.039
	T	-61.15	2.76 × 10^-27^	1.34 × 10^-5^-0.00057	0.026
	DHT	0.21	1.017	1.028-1.484	0.024
Hormonal function	E2	1.14	3.12	1.13-8.64	0.028
	A-dione	-0.019	0.98	0.97-0.998	0.026
Hormonal bother	E2	2.006	7.44	1.82-30.30	0.005
	T	-30.8	4.19 × 10^-14^	4.32 × 10^-24^-0.00041	0.017
	cortisol	0.076	1.08	1.02-1.14	0.008
12Mo					
Sexual bother	E2	1.082	2.95	1.11-7.82	0.030
Hormonal bother	A-dione	-0.0089	0.991	0.983-0.9997	0.042
	DHEA-S	-0.0174	0.983	0.966-0.999	0.049

B: regression coefficient.

PRE: pretreatment 6MO: 6 months after initiation of LH-RH agonist treatment.

12MO: 12 months after initiation of LH-RH agonist treatment.

T: testosterone, DHT: dihydrotestosterone, E2; estradiol, DHEA-S: dehydroepiandrosterone-sulfate.

A-dione: androstenedion, MMSE: mini-mental state examination.

EPIC: expanded postate cancer index composite.

## Discussion

This study examined the changes in cognitive function using the MMSE and in sexual and hormonal functions using the EPIC questionnaire.

We observed no significant worsening in the MMSE scores in this study. The effects of ADT on cognitive functions are controversial. Nelson *et al*. [9] summarized nine studies on this matter, and stated that ADT impaired cognitive functions subtly, especially the visuospatial abilities and executive functioning. Alibhai *et al*. [10] compared the cognitive functions of non-metastatic prostate cancer patients treated with ADT with those of prostate cancer patients not receiving ADT and healthy controls. They observed no consistent evidence of adverse effects on cognitive functions. In a similar study, Mohile *et al*. [11] focused on the preexisting impairment of cognitive functions in elderly subjects and stated that the baseline prevalence of cognitive impairment affected the results. We stratified the patients according to the baseline MMSE scores and found no significant difference during the study. Recently, Chao *et al*. [12] reported a prospective study of the effects of ADT on brain function using functional MRI. In that study, 6 months of ADT clearly impaired brain activations during cognitive control and functional brain connectivity on functional MRI. Interestingly, cognitive function tests showed no significant impairment at this point. Further studies of the association of conventional cognitive function tests with brain function imaging are warranted.

The QOL was assessed using the EPIC questionnaire, which has been validated in Japanese [5]. The summary scores of both sexual and hormonal functions at 6 and 12 months worsened significantly in comparison with those before treatment. Although the sexual functions worsened significantly while on ADT, sexual bother improved during the treatment. This tendency was consistent with the results of the validation study [5]. The exact reason remains unknown; however, the loss of libido might reduce the sexual desire that causes sexual bother. The trends in the hormonal function and bother scores were similar to and consistent with the validation study [5].

In this study, we focused on the hormone levels and QOL assessments and showed the detailed changes in the hormonal domain scores (Tables 3 and 4). Among hormonal functions, hot flashes and body weight gain were worsened significantly on ADT. Only hot flashes were significantly associated with bother. Body weight gain is one of the important adverse events caused by ADT. In the present study, about one-third of all patients experienced body weight gain after 6 months of ADT. Interestingly, the proportions of patients experiencing body weight gains >5 kg or ≤ 5 kg were very similar at 6 and 12 months. Lee *et al*. [13] studied the effect of ADT on body composition changes in prostate cancer patients. On ADT, the fat and lean masses were increased significantly only in patients not receiving ADT. By contrast, a significant loss in bone mineral density occurred in both the patients not receiving ADT or those pretreated with ADT. Our findings regarding body weight changes are consistent with these findings. This information would be helpful when obtaining informed consent from patients who need ADT. Gay *et al*. reported on the QOL assessment using the EPIC questionnaire in patients with prostate cancer treated with neoadjuvant ADT [14]. The sexual and hormonal summary scores were decreased significantly after 2 months of neoadjuvant ADT. In this study, the question items for hormone bother were summarized. The percentage of patients with bother tended to be higher in the cohort of Gay *et al*. [14] in comparison with our study. This might be due to age differences, 70.2 *vs*. 67.5 years, ethnic differences, or tolerance of ADT.

Finally, we investigated the association of cognitive, sexual, and hormone functions with serum hormone levels. In a previous study, we detected a significant decrease in both testicular and adrenal androgens after LH-RH agonist treatment [4]. The decreases in T and E2 were associated with cognitive functions [15]. However, the association of adrenal androgen levels with cognitive or sexual/hormone functions has not been examined. Furthermore, cortisol levels and stress are related [16], so we also examined the cortisol levels. In the cognitive function test, low E2 levels were significantly related to worse MMSE scores in our study. This finding was consistent with Salminen *et al*. [17], who investigated cognitive functions in 23 patients with ADT-treated prostate cancer. The serum E2 levels before and after 6 and 12 months of treatment were correlated with visual memory and verbal fluency. In our study, the A-dione and cortisol levels were associated with the MMSE scores. The odds ratios of A-dione and cortisol were 1.02 and 0.94, respectively, and the role of hormonal changes was unknown. Regarding sexual and hormone functions, low T levels were the factor most significantly associated with worse sexual and hormone bother. High E2 levels were significantly associated with worse sexual bother and both hormone function and bother. In males, E2 is synthesized from T by aromatization in peripheral tissues, including fat [18]. ADT causes a change in body composition, and the fat mass generally increases [13]. In our series, 33.3% of the patients experienced significant body weight gain during the first 6 months of ADT. High E2 levels might affect the QOL changes via this mechanism. High cortisol levels were associated with worse hormonal bother. Cortisol is the most researched stress hormone [16]. Stress during ADT might be associated with high cortisol levels. We observed that low adrenal androgen A-dione and DHEA-S levels were significantly associated with worse sexual and hormonal functions. No relationship between adrenal androgen levels and these functions has been reported. Recently, a new class of CYP17 inhibitor, abiraterone acetate, was approved for treating castration-resistant prostate cancer [19]. Abiraterone acetate significantly reduced the serum adrenal and testicular androgen levels [20]. Further analyses focusing on sexual and hormonal functions are warranted in patients treated with new hormone agents.

This study had several limitations. The first is the small number of patients subjected to the multivariate analysis. Another is the evaluation of cognitive function. We used only MMSE scores, and this score does not cover details of cognitive functions. However, this is the first study to examine the association of testicular and adrenal androgen levels and ADT with cognitive and sexual/hormonal functions. Further large-scale studies are warranted.

## Conclusion

LH-RH agonist monotherapy worsened sexual and hormonal functioning and hormonal bother, but not sexual bother or cognitive function, as assessed by the MMSE and EPIC questionnaires. The observed changes were associated with adrenal and residual testicular androgen levels.
